# Supplementary material for: DNA methylation profiling in recurrent miscarriage
Source: PeerJ. 2020 Jan 7;8:e8196. doi: 10.7717/peerj.8196 (PMC6953351; doi:10.7717/peerj.8196)
Supplement: Supplemental Information 3 [file peerj-08-8196-s003.docx]

1. Experiment description

In the present study, we used the Illumina Infinium HumanMethylation450 array platform to conduct a genome-wide screening of DNA methylation in decidua samples from the products of conception of women with recurrent miscarriage (RM). Furthermore, to gain insight into the molecular regulatory mechanisms of RM, gene ontology (GO) and pathway enrichment analyses were used to explore the potential diagnostic biomarkers and possible regulatory targets.

2. Array design

Microarray analysis was performed using Illumina Infinium HumanMethylation450 BeadChip. An overview of genome-wide DNA methylation array design is available at http://www.genenergy.cn/

3. Samples

We enrolled 15 healthy pregnant women and 15 patients with RM from the outpatient department of Gynecology and Obstetrics, The Second Hospital of Tianjin Medical University, China. All participants were recruited in accordance with the same inclusion and exclusion criteria. The participants were considered to have RM if they had at least two consecutive miscarriages. The participants were considered as controls if they had at least one live birth and no history of miscarriage, still birth, preterm labor, or pre-eclampsia and if their pregnancy was terminated for non-medical reasons or they underwent legal abortions. The exclusion criteria included endocrine diseases, infections, chromosomal abnormalities, immunological diseases, and anatomical abnormalities of the genital tract. Gestational weeks ranged from 6 to 12 weeks, and the maternal age ranged from 22 to 42 years. Decidual tissue was collected anonymously via curettage in The Second Hospital of Tianjin Medical University from June 2013 to August 2013. All collected tissues were fragmented and stored immediately at −80°C in RNAlater solution (Invitrogen, Carlsbad, CA) until RNA and DNA extraction. Written informed consent was obtained from all patients who provided tissue samples, and consent to publish research data derived from these collected samples was also obtained. The collection of the samples for this study was approved by the Shanghai Institute of Planned Parenthood Research Clinical Ethics Review Board.

4. Hybridizations

The protocol and conditions used for hybridization, blocking and washing, including any post-processing steps such as staining: DNA preprocessing and array hybridization were performed according to the manufacturer’s directions. The detailed protocol is available at the following url: https://figshare.com/s/a9d9d5de1c4b530dbd50.

5. Measurements

DNA from each sample was treated with sodium bisulfate and processed for analysis on the Illumina Infinium HumanMethylation450 array platform at Genergy (Shanghai, China). Raw data were submitted to the figshare.

6. Normalization controls

Before proceeding to the statistical analysis, all data were processed via Beta Mixture Quantile dilation. Linear models were developed to calculate P-values using the Limma package of the R software (Smyth 2005). After Benjamini and Hochberg correction, we screened significant methylation variable positions. Meanwhile, using the Probe Lasso method of the ChAMP package, we estimated the differentially methylated regions.
